# Statistical Analysis of Sleep Spindle Occurrences

**DOI:** 10.1371/journal.pone.0059318

**Published:** 2013-04-01

**Authors:** Dagmara Panas, Urszula Malinowska, Tadeusz Piotrowski, Jarosław Żygierewicz, Piotr Suffczyński

**Affiliations:** 1 Institute for Adaptive and Neural Computation, School of Informatics, The University of Edinburgh, Edinburgh, United Kingdom; 2 Epilepsy Research Laboratory, Department of Neurology, Johns Hopkins University School of Medicine, Baltimore, Maryland, United States of America; 3 Department of Psychiatry, Warsaw Medical University, Warsaw, Poland; 4 Laboratory of Biomedical Physics, Institute of Experimental Physics, Faculty of Physics, University of Warsaw, Warsaw, Poland; University of Michigan, United States of America

## Abstract

Spindles - a hallmark of stage II sleep - are a transient oscillatory phenomenon in the EEG believed to reflect thalamocortical activity contributing to unresponsiveness during sleep. Currently spindles are often classified into two classes: fast spindles, with a frequency of around 14 Hz, occurring in the centro-parietal region; and slow spindles, with a frequency of around 12 Hz, prevalent in the frontal region. Here we aim to establish whether the spindle generation process also exhibits spatial heterogeneity. Electroencephalographic recordings from 20 subjects were automatically scanned to detect spindles and the time occurrences of spindles were used for statistical analysis. Gamma distribution parameters were fit to each inter-spindle interval distribution, and a modified Wald-Wolfowitz lag-1 correlation test was applied. Results indicate that not all spindles are generated by the same statistical process, but this dissociation is not spindle-type specific. Although this dissociation is not topographically specific, a single generator for all spindle types appears unlikely.

## Introduction

Spindles were first described in the early XX century as a group of rhythmic waves of increasing and decreasing amplitude, appearing over a background of low voltage EEG or slow waves sleep episodes. Spatial and temporal correlation over the scalp and a distinctive pattern of varying density of occurrences during the night had lead researchers to accept spindles as a hallmark of a separate phase of sleep: stage II [1]. As this phenomenon derives from an early stage of development of EEG and has predominantly been described in terms of visual analysis, the exact parameters to define spindles vary across researchers. According to the classification of Rechtschaffen and Kales [1] these parameters are: frequency within the range of 12–14 Hz; and duration of at least 0.5 sec, so that 6 to 7 distinct waves of oscillation can be observed. Nowadays, however, such a definition is believed to be too restrictive and a wider range of frequencies is usually adopted; for example: 11–15 Hz by Schabus et al. [2], 10–16 Hz by Z?ygierewicz et al. [3]; or 8–15.5 Hz by De Gennaro and Ferrara [4]. As there is no consensus in this area it is difficult to provide a coherent description of spindle properties.

Presently spindles are understood to be a mode of widespread neuronal activity specific to sleep, reflected as spindle-shaped waves in EEG and arising from an interplay between thalamus and cortex. As it has become known over the last two decades, owing to animal studies of M. Steriade (rev. in [5] and [6]), spindle occurrences in EEG correlate with neuronal oscillations in the thalamocortical networks. It transpires that the reciprocal interactions between reticular neuron groups and thalamocortical cell populations produce a spindling activity in the thalamus. This activity is then transferred to the cortex via thalamocortical projections, and reciprocal corticothalamic activation serves to synchronize the spindling activity over the thalamus - and in turn over the cortex itself.

Importantly, spindles are hypothesized to constitute a functionally important feature of sleep. What is generally assumed is that spindle oscillations in corticothalamic networks are the reason for the lack of perceptual awareness and unresponsiveness observed in sleep: the reticular inhibition of thalamus that produces spindles prevents the thalamocortical neurons from responding to ascending input and conveying it onto cortex [6] [7]. Additionally, recent studies suggest that spindles might play an important role in memory consolidation [5] [6] [8] [9]. It is posed that the volleys of spikes constituting spindles promote significant calcium entry into pyramidal cells [5] and both the frequency of oscillations and the pattern of recurrence over stage II sleep are optimized to best induce long-term plasticity [8].

The hypothesis that sleep serves memory consolidation is a hotly debated issue in general [10]. To add to the ambiguities of the particular role of spindles, this oscillation presents a characteristic spectral discrepancy in topographical manifestation. Already in the mid XX century a domination of 12 Hz oscillations over frontal lobes and 14 Hz oscillations over parietal lobes was first reported [11]. Further studies corroborated the existence of two subtypes of spindles [2] [3] [4] [12] [13], differing not only in frequency and location but also varying in reactions to drugs, melatonin level, menstrual cycle or age (rev in [4]; [13]), as well as hemodynamic cerebral correlates [2]. Thus it has become accepted to distinguish two kinds of oscillations: LFS (low frequency spindles), with frequencies centered at around 12 Hz, dominant over frontal lobes and appearing without a distinguishable pattern; and HFS (high frequency spindles), with frequencies centered at around 14 Hz, dominant over parietal lobes and presenting a periodic recurrence of approx. 4 Hz [3]. To date there is no confirmed theory that would explain the separation of spindles into two classes, but it is plausible that spindle variability across regions reflects the underlying anatomical differences. It has been shown in cats that anteroventral and anteromedial thalamic nuclei do not receive inputs from the reticular nucleus [14]. Accordingly, barbiturate induced spindles observed in these nuclei might be a passive process reflecting field potentials actively generated in neighboring thalamic nuclei [14]. Also, Smith et al. [15] reported considerable differences in relative density of GABA-immunoreactive cell bodies and axon terminals across thalamic nuclei in the squirrel monkey. Related to the latter study, there exists an elegant hypothesis based on mathematical modeling [16] which explains non-uniform spindle characteristics in terms of varying synapse densities. It appears that, in a model of interacting reticular and thalamocortical cell populations, the parameter corresponding to average synapse density can influence the resulting spindling pattern in a way very similar to the observed discrepancy between LFS and HFS (i.e. produce lower frequency spindles at random time intervals and higher frequency spindles with a periodic re-occurrence). The hypothesis then poses that two kinds of spindles would result from similar generators, located in anatomically separable networks of different coupling strength between reticular and thalamocortical populations. Because average synapse densities differ between the networks, the resulting dynamics of spindling is different, and both central frequencies and type of statistical process are different: random in frontal and more periodic in posterior derivations.

Altogether, the observations of discrepancies between LFS and HFS has lead some researchers to pose that behind those two types of spindles lie two separate generators [2] [13] [16], and even hint at the possibility of different functional roles of spindles [2] [13]. But to stand a chance of fully understanding the role spindles play in the sleeping brain it is important to better understand the spatiotemporally distinguished different types of spindles. A matter especially calling for attention is the issue of pattern occurrence varying with frequency and location, and the matter of possible separate generators, since in the memory consolidation hypothesis these particular characteristics are a crucial factor [17]. This provides the motivation for the present research.

Our work aims to shed some light on the matter of the dualism of spindles and provide clues as to whether spindles at different topographical locations could indeed stem from separate generators. To achieve that, we set to establish whether the statistical pattern of occurrence differs between them, as predicted by the modelling [16] - namely, whether indeed spindles in frontal location manifest a non-random pattern of appearance, in contrast to spindles in parietal locations. The approach here, inspired by work on epilepsy [18] and adopted from point-process methods, was to fit a gamma distribution to the inter-spindle intervals and - based on the obtained parameters - identify the most likely of the processes behind such a distribution of occurrences. Additional test of serial correlation was also applied to establish whether intervals were independently drawn from the underlying distribution. What transpires from the results is that the statistical behavior of spindles is not consistent topographically nor across subjects. While neither frontal nor parietal spindles can be uniquely associated with either a random or non-random generation mechanism, it is often the case that in a single subject spindles appear to stem from different statistical processes. Therefore a single generator for both spindles types appears unlikely.

## Materials and Methods

### Experimental Data

The study was approved by the Ethics Committee at the Warsaw Medical University. Participants volunteered to take part in sleep research and gave informed written consent.

Overnight EEG recordings of 20 healthy subjects comprise the experimental data used in the study. Acquisition was performed during two consecutive nights; only the results of the second recording are considered in the study. Standard 10–20 system with silver electrodes was employed, as well as supporting EOG and EMG channels. Prior to sampling at a frequency of 128 Hz, an analog bandpass filter (0.5–30 Hz) was applied to the signal. Sleep stages were identified by an experienced sleep researcher based on 20 sec epochs, according to R&K rules [1].

As the analysis aims to confront two types of spindles, four channels expected to demonstrate different spindle types are considered in the present study (chosen on the basis of a high resolution topographical study by Z?ygierewicz et al. [3]). The selected channels are: Fpz, Fz, Cz, and Pz.

To reduce the influence of sleep stage effects on the spindle generation only spindles produced during stage 2 sleep were considered.

### Automated Spindle Detection

With the amount of data in the study (20 subjects, 4 channels of approx. 8 hour sleep), employing automated detection of sleep spindles was necessary. In order to best identify the spindles a processing based on Matching Pursuit (MP) algorithm was adopted: signal was decomposed into a set of functions, and structures corresponding to spindles were then identified.

#### Matching pursuit

Matching Pursuit (MP) is a time-frequency analysis method introduced by Mallat and Zhang [19] that implements a suboptimal adaptive approximation of a signal. MP algorithm iteratively decomposes a signal into a set of functions (called *atoms*) chosen from a redundant basis (called *dictionary*) on the basis of highest inner product fit. The dictionary consists of a basis of sinusoids, a basis of Dirac deltas and a redundant set of Gabor functions (Gaussian modulated with cosine). The atoms are chosen from the dictionary in an iterative algorithm. In each step the atom *f_i_* that gives the highest inner product (here denoted as <x,y>) with the current residuum *R^i^* is selected, as follows:
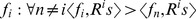
(1)and the next residuum is obtained as:



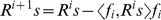
(2)The resulting signal representation is an orthogonal set of atoms that, provided the dictionary is complete, is convergent to the signal:
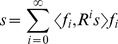
(3)


In this paper an implementation of the MP algorithm with stochastic dictionary structure developed at the Department of Biomedical Physics, University of Warsaw, was used [20].

#### Identification of Spindles

A single spindle can be well approximated by a single Gabor function. It is assumed here that spindles can be identified in the MP decomposition of a signal as Gabor atoms with the following constraints on the parameters (as in [3] and [21]):

frequency in the range 10–16 Hz;
duration 0.5–2 sec;
amplitude over 15 µV.

#### Amplitude–Frequency plots

**Figure 1:**
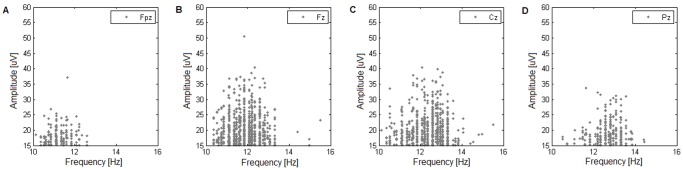
Example amplitude-frequency scatter plot. Spindle properties of subject 3: amplitude-frequency plots for four scalp locations (**(A)** frontal Fpz, **(B)** frontocentral Fz, **(C)** central Cz, and **(D)** parietal Pz; according to the 10–20 electrode placement standard). Upon progressing from channel Fpz to Pz, the mean frequency of the cluster of spindles shifts from approx. 11 to 13 Hz.

Scatter plots of amplitude versus frequency of the identified spindles were employed in order to characterize properties of the spindles in each subject. On a topographical set of such plots (Fig. 1) we visualize the amplitude-frequency distribution of spindles, which provides a rough scalp localization of spindles of given frequency.

### Statistical Analysis of Spindle Occurrences

To determine the underlying statistical process of spindles generation an approach adapted from point-process analysis methods was used here - maximum likelihood estimation [22] of gamma distribution parameters best fitting the experimental data, i.e. the distribution of inter-spindle intervals (inter-spindle intervals were taken as distance in time between the centers of identified spindles).

#### Gamma distribution

Probability density of a gamma distribution is given as:
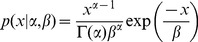
(4)where Γ is the gamma function. Parameters α and β, named shape and scale parameters, both determine the overall shape of the distribution, yet the first of these is responsible for the location of the maximum, being either at the origin for α≤1 or at nonzero value for α >1.

Gamma distribution arises naturally in processes for which the waiting times between Poisson distributed events are considered. Therefore it is often used as a model probability to describe distribution of waiting times of random variables observed in nature, also in neuroscience - e.g. to model inter-spike intervals of spiking neurons [23] [24] and inter-seizure intervals in epilepsy [18] [25]. Following Maimon and Assad [24], the value of the shape parameter, α, may be a useful measure of inter-event regularity. For α = 1, gamma distribution reduces to an exponential distribution, suggestive of a Poisson process. When α >1, the distribution is unimodal, suggestive of increased regularity from Poisson. When α <1, the distribution has an excess of very short intervals and a deficiency of very long ones, suggestive of increased irregularity from Poisson. Therefore a gamma distribution may provide useful information regarding the type of deviation from the Poisson process towards more periodic behavior or towards more irregular one, and as such it was an excellent candidate for the purpose of this study.

**Figure 2:**
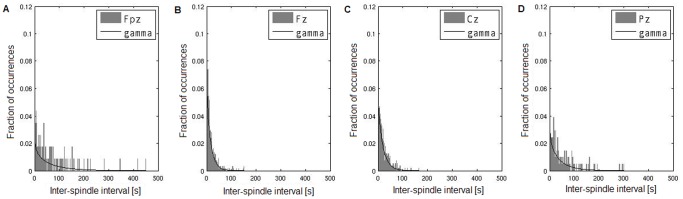
Example overview of statistical spindle properties. Statistical properties of spindles in subject 3: in grey, histograms of inter-spindle intervals for four scalp locations (**(A)** Fpz, **(B)** Fz, **(C)** Cz, and **(D)** Pz; inter-spindle interval taken as center-to-center separation in time of automatically detected spindles of stage 2 sleep; bin width of histograms: 1s); superimposed in solid lines, gamma distributions fit to the distribution of inter-spindle intervals. In all four locations, the maximum likelihood distribution fit yields a gamma distribution with shape parameter close to one, suggesting a Poisson process.

The maximum likelihood parameter estimation was performed with the use of *gamfit* procedure offered by the Statistic Toolbox of the Matlab program (Matlab, The Math Works Inc., Natick, MA, USA). The procedure returns the maximum likelihood estimates of the parameters and their 95% confidence intervals. Example histograms of inter-spindle intervals and fitted distributions are shown in Fig. 2.

#### Evaluating the distribution fitting

**Figure 3:**
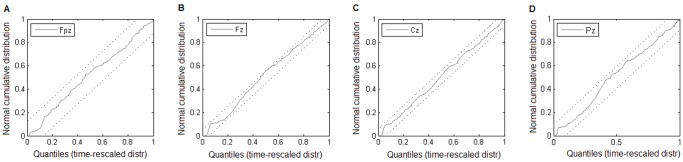
Example of gamma fitting evaluation. Quality assessment of gamma distribution fits for subject 3 (the fits from Fig. 2) across locations (**(A)** Fpz, **(B)** Fz, **(C)** Cz, and **(D)** Pz): Kolmogorov-Smirnov plots for each of the four fits of gamma distribution to the spindle interval distribution; dotted lines represent 95% confidence bounds. In all four panels the KS plots lie entirely within the confidence bounds pointing to statistically acceptable agreement between the model and the data.

To evaluate the quality of the fit, the Kolmogorov-Smirnov plot was used. KS plot is a graphical method similar to a quantile-quantile plot, but instead of comparing the fitted distribution against the empirical one it uses rescaled time occurrences and compares them against the cumulative distribution function of a normal Gaussian. The time occurrences were rescaled according to the formula:
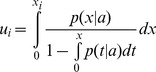
(5)where *{x_i_}* denotes a set of events and *p(x|a)* denotes the probability density function (with *a* standing for any parameters; here, the parameters α and β)_._ As follows from the time rescaling theorem [26], such a transformation yields a set of values from an exponential distribution, if only *{x_i_}* are subject to *p(x|a)*. A further transformation:
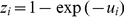
(6)should produce values from a uniform distribution. For each of the analyzed channels the cumulative distribution function of a uniform distribution was plotted against the ordered zi values, thus forming a KS plot. 95% confidence bounds were constructed as *i*±(*1.36*)/*N* *^1/2^*, according to [26]. Example of such a plot is shown in Fig. 3.

In the case of the KS plot trace transgressing the confidence bounds, the corresponding gamma distribution fit was deemed unreliable and discarded from further consideration.

#### Additional non-parametric test for randomness

Using a distribution of inter-event intervals for statistical inference means that effectively all serial information about the time series is ignored. This presents a problem, since any interpretation of a gamma fit rests on the assumption that the intervals underlying the empirical distribution are independent. Therefore, to ensure the interpretability of the gamma fits, an additional test was employed to establish whether successive inter-spindle intervals were drawn at random from the underlying distribution: a non-parametric serial correlation test proposed by Wald and Wolfowitz [27].

Lag-1 serial correlation coefficient was used as the test statistics:
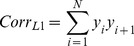
(7)(where *y_i_* is an inter-spindle interval). An exact non-parametric test would require computing a serial correlation coefficient for all possible permutations of the intervals in order to obtain the test probability distribution. However, with as many as several hundreds of spindles in some patients, such a procedure is computationally infeasible. Therefore a resampling approach was adopted here, with a fixed number of random permutations *N_p_.*

To establish a sufficient level of sampling, the procedure was repeated for *N_p_* = 10^4^, 10^5^, 10^6^, and 10^7^ and the resulting probability estimates were compared. Beyond 10^6^ permutations the p-values changed by less than 0.5%, therefore *N_p_* = 10^7^ was deemed reliable.

## Results

### Intersubject Variability

What needs to be noted first is the extent of inter-subject variation of spindles characteristics. The numbers of identified structures range from twenty to approx. a thousand spindles occurring in a single channel overnight (Fpz channel, subjects 8 and 16, respectively). Considerable differences manifest also between individual frequency ranges: in some cases observable spindles do not exceed 14 Hz (Pz channel, subject 1) whereas for some subjects practically all the spindles fall between 12 and 16 Hz (Pz channel, subject 4). Importantly, the distributions of frequencies across channels vary greatly across participants of the study (as described in the next paragraph).

### Amplitude-frequency Distributions and Topographical Location

**Figure 4:**
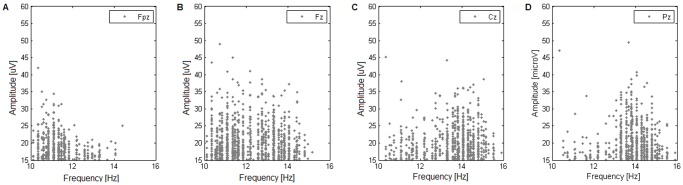
Subject with clear topographical separation of spindles. Amplitude-frequency plot of an example subject from the group that exhibits a clear topographical separation of low and high frequency spindles: in the frontal channel, **(A)** Fpz, most spindles do not exceed 12 Hz, with nearly no activity over 14 Hz; in the parietal channel, **(D)** Pz, most spindles fall between 13 and 16 Hz, with only a few appearing below 12 Hz.

**Figure 5:**
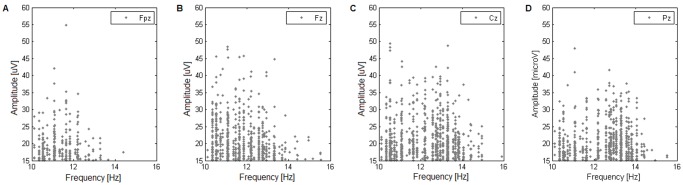
Subject with topographically restricted HFS. Amplitude-frequency plot of an example subject from the group that exhibits topographically restricted high-frequency spindles, and non-restricted low-frequency spindles: in the frontal channel, **(A)** Fpz, most spindles appear below 13 Hz; in the parietal channel, **(D)** Pz, spindles both below and above 13 Hz are common.

**Figure 6:**
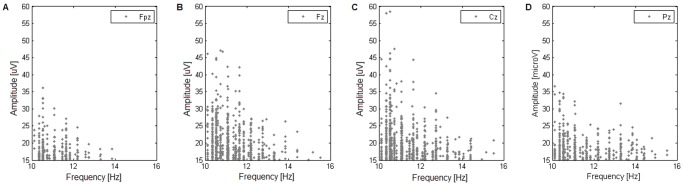
Subject without a clear topographical distinction of spindles. Amplitude-frequency plot of an example subject from the group that exhibits no clear topographical trends: the distribution is similar across scalp locations, with a dominance of low-frequency spindles and a very faint presence of high-frequency spindles over all channels.

Visual analysis of frequency-amplitude distributions reveals that the subjects can be roughly classified into three categories:

- distributions of spindle frequencies show a presence of topographically restricted LFS and HFS (Fig. 4); from the 20 subjects included in the study only 5 exhibit a clear separation into frontally located LFS and parietally located HFS (subjects 3, 4, 5, 19, 20); a further 7 exhibit some indications of separation (subjects 1, 2, 8, 11, 12, 15, 16);
- distributions of spindles indicate that LFS are present across all channels with only HFS being topographically restricted (Fig. 5); 5 such subjects can be identified (subjects 6, 7, 10, 13, 18);
- distributions of spindle frequencies display no clear separation of spindles nor clear topographical trends (Fig. 6); there are 3 such subjects (subjects 9, 14, 17).

### Distribution Fitting Results - Dominance of a Poisson Process

As explained in the Methods section, in order to assess the statistical properties of spindles in different topographical locations a gamma distribution was fit to the distribution of spindle occurrences for each subject in four channels (Fpz, Fz, Cz, and Pz). KS plots were then used to discount statistically unsound fits; there were 17 such cases, mostly channels with too few spindles - and those were discarded from further consideration.

**Figure 7:**
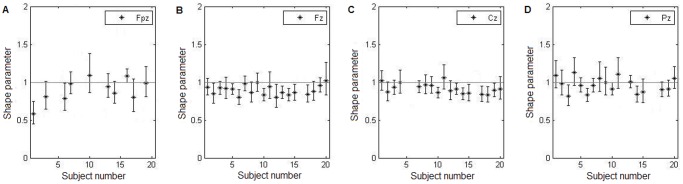
Fitted shape parameter across locations. Values of the shape parameter of fitted gamma distribution for each subject; each panel represents a different scalp location (**(A)** Fpz, **(B)** Fz, **(C)** Cz, and **(D)** Pz); unreliable fits (as assessed by the KS plot) were removed; bars denote 95% confidence bounds of fitted parameter. Shape parameter reflects the statistical nature of the generation process and, as can be seen above, most channels and most subjects exhibit hallmarks of a Poisson random process (shape parameter not different than 1).

The shape parameter of fitted gamma distributions in all subjects and channels stays within the bounds of [0.55 1.66], which is a fairly compact range. After excluding the fits deemed unreliable this range further narrows to [0.55 1.14]. In 42 out of 65 cases the 95% confidence bounds of the shape parameter of fitted gamma distribution include the value of 1, suggestive of a Poisson process of generation. In the remaining 23 cases, the shape parameter is lower than 1 as the 95% bounds do not reach the threshold value of 1, suggestive of increased irregularity from Poisson. Overall there is no indication of a distinct statistical difference between the channels, although Fpz and Pz seem to contain less deviations from a Poisson process and exhibit a wider variance within the shape parameter values when compared to Fz and Cz (Fig. 7).

### Is there a link between Apparent Discriminability of HFS/LFS and Statistical Properties of Spindle Distributions?

As reported before [3], the LFS in frontal channels appear to occur randomly, while the HFS in parietal channels exhibit a periodic pattern of appearance. Accordingly, we inspected the values of shape parameters in all subjects as a function of their topographical location.

**Figure 8:**
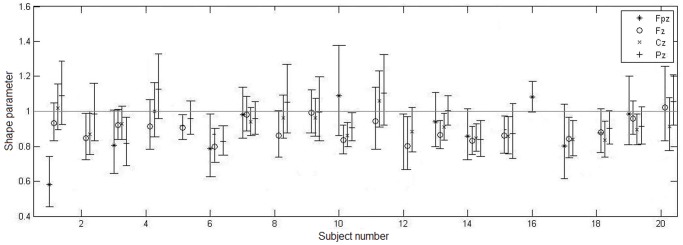
Fitted shape parameter across subjects. Values of the shape parameter of fitted gamma distribution for each of the scalp locations, assembled subject-wise; unreliable fits (as assessed by the KS plot) were removed; bars denote 95% confidence bounds of fitted parameter. There is no consistent effect of location on the shape parameter that would be exhibited across subjects and that would point to two distinct statistical processes governing frontal and parietal spindles.

As can be seen in Fig. 8, subjects 1, 4, 8 and 11 exhibit a common tendency of statistical properties: the value of the shape parameter progressively increases as location approaches parietal regions. It appears that in the Fz channel the generation process is random, while in the Pz channel the generation process exhibits increased regularity from Poisson. However, only those four subjects from the total of 20 in the study show such tendencies. Within the remaining 16, even among subjects whose amplitude-frequency plots indicate a presence of two types of spindles, there is no reliable indication of topographical differences in statistical properties. In fact, in subjects 7, 14 and 19, the shape parameter in the parietal derivation is slightly lower than in the frontal derivation.

### Serial Dependencies and Topographical Location

As mentioned in the Methods section, statistical analysis of a distribution of inter-spindle intervals ignores the ordering of intervals and therefore ignores any serial relationships in the data. This is an important issue in the case of the results reported here, because the interpretation of gamma fits being consistent with a random process rests on the assumption of intervals being independent of each other.

The results of the serial correlation test indicate that only 54 out of 80 series of intervals are random (on a significance level of 0.05, two-sided test). This is interesting in the context of the two possible generators of spindles under investigation, as these results seem to show some topographical tendencies that would differentiate most frontal spindles from more central and parietal ones. In Table 1 the summary of performed test is provided.

**Table 1:** Serial correlation test results.

	Fpz	Fz	Cz	Pz
Subject 1	0	0	0	0
Subject 2	0	0	0	0
Subject 3	0	0	0	0
Subject 4	0	1	0	1
Subject 5	1	1	0	1
Subject 6	0	1	1	1
Subject 7	0	1	1	1
Subject 8	0	0	0	0
Subject 9	0	1	0	0
Subject 10	0	1	1	1
Subject 11	0	0	1	0
Subject 12	0	0	1	0
Subject 13	0	1	1	1
Subject 14	0	1	0	1
Subject 15	0	0	1	0
Subject 16	0	1	1	1
Subject 17	0	0	0	1
Subject 18	0	0	0	0
Subject 19	0	1	0	0
Subject 20	0	0	0	0

Results of Wald-Wolfowitz serial correlation test. 0 indicates that the null hypothesis cannot be rejected, i.e. the intervals are random and independent; 1 indicates rejection of the null hypothesis, i.e. there is indication of serial dependence.

In subjects 1, 2, 3, 8, 18 and 20 randomness cannot be rejected across all channels. However, only Fpz derivations seem to generally comply with the criteria of randomness; the null hypothesis is rejected only in 1 out of 20 cases. At other locations, the hypothesis is rejected in roughly half number of cases. Therefore it might be concluded that spindles recorded frontally and those recorded over the centroparietal cortex may have different statistical properties.

## Discussion

The analysis conducted in the present study shows that spindles are a very inhomogeneous phenomenon with widely varying characteristics, which adds to already existing ambiguities concerning detection and analysis of these oscillations. Hence it is difficult to make solid inferences regarding the common spindles properties. Below we present a tentative interpretation of obtained results and how this may bear on the current view on spindles.

Firstly, it appears that the spatial separation of spindles of low- and high-frequency bands is not a universal characteristic of spindles, as presented in section 3.2. While in some subjects LFS remain constrained to frontal areas and HFS dominate over parietal areas of scalp, in others there is no such clear topographical differentiation.

It is certainly possible that this is due to acquisition and detection issues. The cutoff amplitude of 15 µV may be excluding from analysis small amplitude spindles in some subjects; and predefined spindle parameters might allow for inclusion of some artifact spindles. But most importantly, due to shortcomings of the data acquisition technique, actual cortical spindle-related activity does not register faithfully at the EEG level. As shown in studies using EEG and MEG simultaneously [28], neither of those recording modalities is reliable, and spindles can occur in one without the accompanying synchronous oscillation in the other. This issue is important when considering any inferences about spindle activity from pure EEG analysis, as most likely EEG is under-sampling spindles, excluding certain signal sources due to the geometry of the cortex.

However, one could expect that such issues would affect all subjects similarly and introduce a systematic bias rather than under-sample different types of spindles in different subjects. Therefore it is our view that spindles present a considerable heterogeneity across subjects - not only in the peak frequencies or the numbers of spindles, but also in the types of topographical distributions. This is substantiated by results such as those of [13]. The authors observe that in different subjects, power spectra exhibit varying degrees of spatial differentiation: for instance in some cases high frequencies (corresponding to HFS) appear in frontal areas with similar power as in the posterior; while in other subjects those frequencies are restricted to parietal and central derivations and are significantly diminished in power in the frontal region. De Gennaro et al. [29] also report vast heterogeneity of spindle properties across subjects. In their sleep EEG study each subject has a different topographical distribution of power spectra in the spindle frequency range and, similarly to our results, they report that only some subjects exhibit a clear presence of two spindle types.

As the spatial separation of spindles of low- and high-frequency bands is not clear-cut, we explored alternative hypothesis regarding the dualism of spindles and examined whether spindles at different topological locations share the same or different statistical properties. Such a question is directly linked to the probable anatomical differences between spindle generators in various parts of the thalamus [14] [15] [16]. To assess what statistical processes lie behind spindles in different locations, a gamma distribution was fit to the distributions of spindle occurrences. The shape parameter of the reliable fits was used to identify whether the generating process is a Poisson process, or exhibits deviation towards increased or decreased regularity from Poisson. Next, the randomness was further examined with a non-parametric test of serial correlation.

Results show that, in general, the distributions of inter-spindle intervals are very well described as gamma distributions as confirmed by a goodness-of-fit KS test. However, the variation of the shape parameter does not present significant topographical tendencies (Fig. 7). The values in all four chosen locations are centered around 1 and in most cases suggest a Poisson process underlying the spindle generation. On comparison, channels Fz and Pz both exhibit very similar statistical behavior and there is no observable overall bias towards a regularity in spindle occurrences, i.e. shape parameter larger than 1, in the posterior areas (as was suggested by Żygierewicz et al. [16]). Interestingly, in four subjects (1, 4, 8 and 11) there might be a trend in the value of the shape parameter having lower value at frontal locations and progressively increasing towards parietal regions. However the proportion of subjects showing such tendency is small (four out of 20). Besides, the 95% confidence bounds of the shape parameter exclude the value of 1 in only one case (electrode Fpz in subject 1) while in all remaining cases the confidence bounds include the unity. Accordingly, the topographically ordered deviation from a Poisson process cannot be put in evidence. Taken together, these results suggest that spindles are generated predominantly in random fashion and the deviations from that rule are not stereotyped topographically.

However, an additional test for randomness calls into question the prevalence of a random process exhibited by gamma fitting results. Results of the serial correlation test uncover that in 14 out of 20 subjects there is at least one EEG channel in which spindle intervals do not appear to be independent and therefore does not correspond to a Poisson process. Although it may be difficult to identify clear topographical stereotypy, it may be noted that in the most frontal derivation Fpz there is only one subject exhibiting significant serial correlation suggesting non-random spindle occurrence. In other derivations non-randomness is significantly more prevalent (Table 1).

Given the results of the serial correlation test a common generator of spindles seems unlikely. Only in five out of 20 subjects do the statistical properties of spindles seem to be spatially uniform. In the remaining cases, a hypothesis of topographically different spindle generators subject to different statistical processes appears to be the case. Interestingly, in the most frontal locations spindles seem to correspond predominantly to a random Poisson process as predicted by the modeling work of Z?ygierewicz et al. [16].

Altogether, based on the spatiotemporal dynamics of spindles, the appealing interpretation is that they cannot be collectively attributed to two separate and statistically distinct processes; nor do they share a common one. In some subjects the spindles manifest uniformly over the scalp, while in a majority of them there are certain differences between locations. We hypothesize that the manifestation over the scalp is a result of an interplay of many individual traits, which is why it is not uniform across subjects. This hypothesis corroborates with recent research into the relationship between spindle activity and general cognitive and learning abilities [30] [31]. Several studies to date relate spindle activity to general intelligence, and Schabus et al. [31] propose that the underlying reason for this relationship is that spindle activity reflects the quality of thalamocortical connections. In light of such suggestions, our findings could be interpreted to reflect varying topography of thalamocortical projections between individuals and the heterogeneity we observe could reflect varying cognitive traits. However, the answer is beyond the scope of current study, as the EEG traces were the only data collected.

In summary, our study suggests that simple statistical properties of spindles may offer an additional marker of spindle heterogeneity and could be used to investigate further the functional role of spindles.
